# Roles of Two-Component Systems in *Pseudomonas aeruginosa* Virulence

**DOI:** 10.3390/ijms222212152

**Published:** 2021-11-10

**Authors:** Maria Sultan, Rekha Arya, Kyeong Kyu Kim

**Affiliations:** Department of Precision Medicine, Graduate School of Basic Medical Science (GSBMS), Institute for Antimicrobial Resistance Research and Therapeutics, Sungkyunkwan University School of Medicine, Suwon 16419, Korea; mariasultantanoli26@gmail.com

**Keywords:** *Pseudomonas aeruginosa*, virulence, two-component system (tcs), biofilm, motility, pyocyanin, cytotoxins

## Abstract

*Pseudomonas aeruginosa* is an opportunistic pathogen that synthesizes and secretes a wide range of virulence factors. *P. aeruginosa* poses a potential threat to human health worldwide due to its omnipresent nature, robust host accumulation, high virulence, and significant resistance to multiple antibiotics. The pathogenicity of *P. aeruginosa*, which is associated with acute and chronic infections, is linked with multiple virulence factors and associated secretion systems, such as the ability to form and utilize a biofilm, pili, flagella, alginate, pyocyanin, proteases, and toxins. Two-component systems (TCSs) of *P. aeruginosa* perform an essential role in controlling virulence factors in response to internal and external stimuli. Therefore, understanding the mechanism of TCSs to perceive and respond to signals from the environment and control the production of virulence factors during infection is essential to understanding the diseases caused by *P. aeruginosa* infection and further develop new antibiotics to treat this pathogen. This review discusses the important virulence factors of *P. aeruginosa* and the understanding of their regulation through TCSs by focusing on biofilm, motility, pyocyanin, and cytotoxins.

## 1. Introduction

Antimicrobial resistance (AMR) has become a serious global health threat because the rapid emergence of AMR has led to considerable increase in morbidity and mortality across the world [1,2,3,4]. Consequently, it is critical to develop new antibiotics or alternative therapeutic strategies to address pathogen antimicrobial resistance [5,6,7]. The increase of multi-drug resistant (MDR), pan-drug resistant (PDR), and extensively drug-resistant (XDR) isolates of *P. aeruginosa* constitute a substantial therapeutic challenge [8]. *P. aeruginosa* has been included in the ESKAPE (*Enterococcus faecium*, *Staphylococcus aureus*, *Klebsiella pneumoniae*, *Acinetobacter baumannii*, *P. aeruginosa*, and *Enterobacter* spp) pathogen list and identified as a critical priority pathogen [9,10]. According to the US Centers for Disease Control and Prevention, 32,600 cases and 2700 deaths caused by multi-drug resistant *P. aeruginosa* were recorded in 2019 [11].

*P. aeruginosa* is a Gram-negative, environmental pathogen that is present in diverse habitats [12]. *P. aeruginosa* is a clinically and epidemiologically important bacterium that causes both acute and chronic infections. Furthermore, this opportunistic pathogen is linked most often to infections in immunocompromised patients [13,14]. The pathogenesis of *P. aeruginosa* depends on the virulence factors, which have a crucial role in bacterial colonization, and on host tissue invasion, which can result in life-threatening infections. The important virulence factors of *P. aeruginosa* include biofilm formation, motility (pili, flagella), pigment (pyocyanin), cytotoxins, phospholipases, elastases, and proteases [15].

*P. aeruginosa* has both resistance and virulence traits, and the versatility is related to its large genome and core-essential genes [16,17]. Its genome is comprised of multiple two-component systems (TCSs) and several regulatory genes [18]. TCSs function via a signal-response coupling mechanism that allows bacteria to recognize and respond to the signals in a diverse environment. A previous report has suggested that various TCSs are involved in regulation of virulence factors of *P. aeruginosa* [19]. It is important to understand how *P. aeruginosa* senses and responds to environmental stimuli via TCSs throughout the infectious process to enhance our knowledge of its pathogenesis. Therefore, it is essential to understand the role of TCSs in virulence to develop novel therapeutics against AMR strains of *P. aeruginosa*. Moreover, these diverse TCS-based control mechanisms of virulence factors shape the adaptation and survival of *P. aeruginosa* in unfavorable environments.

**Figure 1:**
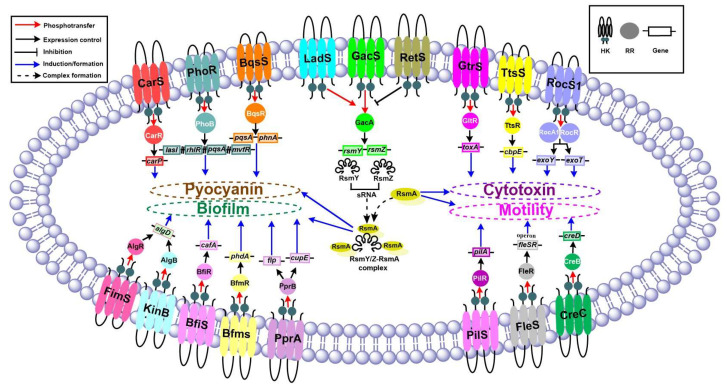
Schematic representation of TCSs that regulate four important virulence factors of biofilm formation, motility, pyocyanin, and cytotoxins, in *P. aeruginosa*. The important TCSs and the genes responsible for producing the respective virulence factors are highlighted. Sensor HKs are shown in the cell membrane (different colors), while their respective round structures signify cognate RRs. The small rectangles represent the genes. The arrows and dotted lines represent various functions as indicated in the inset.

Consequently, it is imperative to review recent advancements in the field of TCSs. This knowledge will help refine our understanding of the intricate regulatory network architecture that controls the virulence of *P. aeruginosa.* In this review, we explore the regulation of four important virulence factors: biofilm formation, motility, pyocyanin, and cytotoxins, by assessing various TCSs of *P. aeruginosa*, as depicted in Figure 1. However, in this review, input signals to TCSs have not been included nor mentioned in figures and text, since the focus has been placed mainly on cellular signaling through TCS and their effects on key virulence factors.

## 2. TCSs in P. aeruginosa

**Figure 2:**
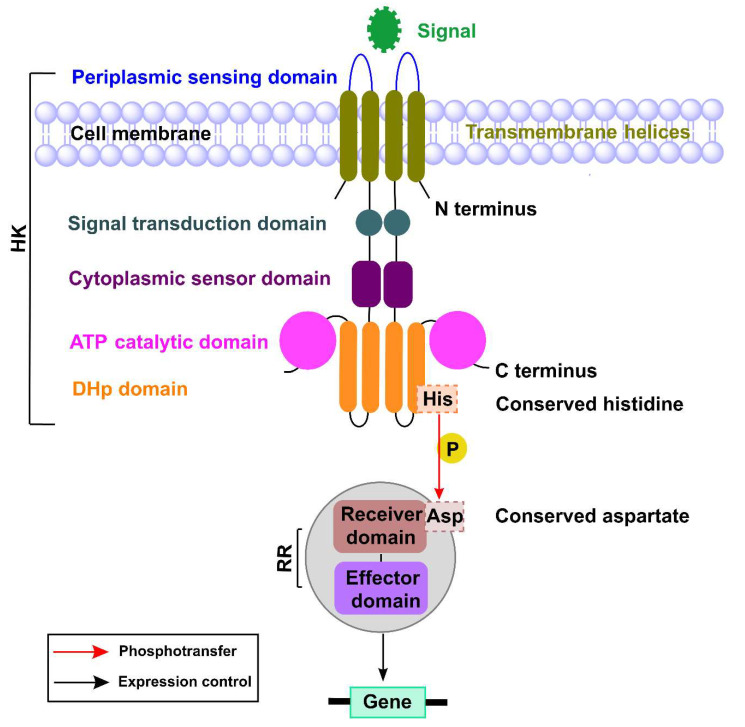
The basic mechanisms of a two-component signaling system. The sensor HK senses the environmental signal using the sensing domain that is connected to the signal transduction domain, a cytoplasmic sensor domain, an ATP catalytic domain, and a dimerization histidine phosphotransfer domain (DHp). The conserved histidine residue of HK transfers the phosphate to the conserved aspartate residue of the receiver domain of its RR. The effector domain of the phosphorylated RR binds to its target and regulates gene expression.

TCSs are key mediators of signal transduction in bacteria [20]. They also play an important role in sensing various external stimuli and responding to changes in environmental conditions. These TCSs are comprised primarily of a histidine kinase (HK) and its cognate response regulator (RR) that governs various signal transduction pathways [21]. The sensor histidine kinases are multidomain structures primarily composed of a periplasmic sensing domain that is responsible for identifying specific signals; the signal transduction domain; cytoplasmic sensor domain; adenosine triphosphate (ATP) catalytic domain; and dimerization histidine phosphotransfer domain (DHp) [21,22]. The sensor HK is responsible for autophosphorylation of the conserved histidine within the HK domain, and it transfers the phosphate from its conserved histidine residue to the aspartate residue of its cognate RR. The RR is responsible for intracellular responses, for which its effector domain undergoes a conformational change that allows it to bind to DNA, which triggers changes in gene expression [21] (Figure 2).

In general, bacterial genomes have a diverse number of TCSs, and the overall number can differ from one bacterium to another [23]. Most bacterial species require an array of TCSs because of the varying input detection domains of HK, which allow the bacteria to receive multiple environmental stimuli. More than half the known TCSs are responsible for controlling virulence factors. The number of TCSs associated with virulence in *P. aeruginosa* is increasing rapidly due to the development of whole genome-based approaches [19]. When comparing the genome sizes of various bacteria, the *P. aeruginosa* PA14 strain has the largest sequenced genome of 6.54 million base pairs (Mbp) and 5973 genes, which is slightly larger than that of *P. aeruginosa* PA01, which has a genome size of 6.26 Mbp [24]. The genome of *P. aeruginosa* PA14 is larger than those of other known pathogenic bacteria such as *Escherichia coli* K12 (4.64 Mbp), *Klebsiella pneumoniae* HS11286 (5.33 Mbp) and *Acinetobacter baumanni* HX386 (4.09 Mbp) [25,26,27]. The number of TCS of *P. aeruginosa* is also higher than other Gram-negative bacteria; such as *P. aeruginosa* PA01 (63 HKs; 64 RRs), *Escherichia coli* K12 (28 HKs; 32 RRs), *Klebsiella pneumoniae* HS11286 (32 HKs; 32 RRs), and *A. baumanni* XH386 (14 HKs; 15 RRs) [18,23,28]. The detailed comparison is mentioned in Table 1.

## 3. Virulence of *P. aeruginosa*

*P. aeruginosa* infection involves a series of stages starting from bacterial adherence and followed by colonization, invasion, dissemination, and finally severe systemic diseases. Its pathogenesis is determined by multiple virulence factors [29]. *P. aeruginosa* infection involves a series of steps, from bacterial adherence followed by colonization, invasion, and dissemination, which finally cause severe systemic infection [29]. In short, adhesion and colonization are the first two important steps of pathogenesis where the bacteria undergo initial attachment to the surface for colonization. In the third step of pathogenesis, the colonized bacteria start to invade the host tissues. *P. aeruginosa* then enters the fourth step for facilitating dissemination and causing systemic infection by damaging host tissues such as skin, blood, respiratory, and urinary tract. Each stage of infection is highly influenced and controlled by multiple virulence factors.

These virulence factors allow bacteria to escape from host defenses and trigger a variety of diseases such as respiratory, skin, blood, and urinary tract infections. 

## 4. Key Virulence Factors in *P. aeruginosa*

The pathogenic profile of *P. aeruginosa* is connected to multiple virulence factors including, but not limited to, a protein secretion system, biofilm formation, cell surface components, quorum sensing system (QS), and exoenzymes, which play an important role in infection severity [30]. Various secreted products such as toxin, exopolysaccharide, and enzymes and cell surface components such as capsules, lipopolysaccharides, glycoproteins, and lipoproteins play a major role in pathogenesis [31]. Moreover, siderophores including pyochelin and pyoverdine are crucial virulence factors that allow bacteria to divide in the presence of ferrous ions and enhance bacterial metal resistance [32]. The Type III secretion systems (T3SS) of *P. aeruginosa* produce various toxins such as ExoU, ExoS, ExoT, and ExoY, which are responsible for ventilator-associated pneumonia [33]. ExoS and ExoT are hetero-bifunctional cytotoxins with amino-terminal Rho-GAP (GTP binding protein of *rho* - GTPase activating protein) and C-terminal ADP-ribosyltransferase activities, respectively. They play a role in interfering with the signal transduction in the host involved in phagocytic oxidation of NADPH (nicotinamide adenine dinucleotide phosphate) [34,35]. Moreover, ExoU is responsible for destruction of cellular physiology through its phospholipase activity [36]. ExoY is another important toxin that has a role in pathogenesis of *P. aeruginosa* based on adenylate cyclase activity [37]. These virulence factors are present in other pathogenic bacteria as well; however, several virulence factors such as pyocyanin, rhamnolipids, and cup fimbriae are specific to pseudomonas species [19,38,39]. Among various virulence factors in *P. aeruginosa*, biofilm formation, motility, pyocyanin, and secreted toxins are the four most important since they are responsible for acute and chronic infections. Although there are two types of regulation systems, TCS and a QS, which control the expression of these virulence factors, we focus on TCSs and their roles in controlling the four key virulence factors in *P. aeruginosa* (Table 2).

### 4.1. Biofilm, a City of Microbes

*P. aeruginosa* cells are highly organized and are able to form a complex community called a “biofilm” or “a microbe city” [80]. The biofilm is enclosed inside the extracellular matrix and can adhere to both biotic and abiotic surfaces. The matrix is composed primarily of lipids, polysaccharides, and extracellular DNA (eDNA) [81]. Biofilm formation is an important trait attribute to chronic *P. aeruginosa* infections which allows the bacteria to evade the host immune response [82,83]. *P. aeruginosa* produces a robust biofilm that is one of the most critical virulence factors in its pathogenesis. *P. aeruginosa* is linked to device-associated infections characterized by formation of thick biofilms on the surface of implanted materials [84].

**Figure 3:**
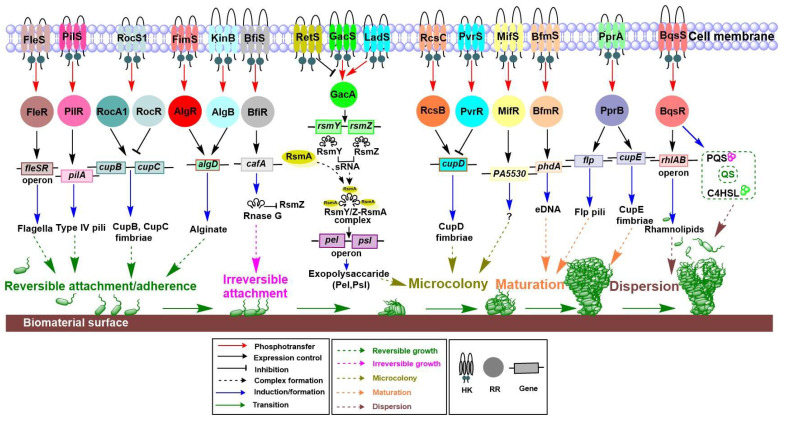
The role of TCSs in biofilm formation. Important stages of *P. aeruginosa* biofilm formation are reversible attachment/adherence, irreversible attachment, microcolony formation, maturation, and dispersion. Many TCS are important for initial adherence. FleSR and PilSR are responsible for flagella and pili motility structures during initial attachment. RocS1-RocR and RocA1 play a role in production of the CupB and CupC fimbriae structure. FimS-AlgR and KinB-AlgB are responsible for alginate biosynthesis and help with adherence. During the initial stages, BfiSR has a role in irreversible growth. The GacSA acts in a parallel and antagonistic manner to LadS and RetS. The free RsmA regulatory proteins, together with sRNAs (RsmY and RsmZ) lead to the complex formation of RsmY/RsmZ-RsmA and play a role in the production of exopolysaccharides Pel and Psl. RcsCB and PvrSR are important for the CupD fimbriae structure. BfmSR and MifSR are essential for regulating microcolony formation and initial maturation during biofilm formation. PprAB is crucial for the CupE fimbriae structure and helps microcolonies to form and mature. BqsSR plays a role in the production of rhamnolipids and is responsible for dispersion of biofilm.

The development of a biofilm of *P. aeruginosa* occurs in five important phenotypic stages (Figure 3). The first step is initial adherence or reversible attachment, in which the free-living bacteria attach to appropriate surfaces, but can also be detached depending on the concentration of bacteria or environmental changes, such as in physical force. In the second step (irreversible attachment), bacteria can be attached irreversibly by lying flat along the surface to protect themselves from physical barriers [85]. After irreversible attachment, the bacteria form small colonies in the extra polymeric substance matrix. Next, microcolonies of the bacteria enlarge and converge with other microcolonies to produce an additional organized phenotype in a non-colonized space. A surface devoid of attached bacteria is filled by reproducing bacteria, which ultimately cover the entire surface to provide a mushroom-like appearance. Finally, in unfavorable conditions, the synthesis of matrix compounds declines, and the matrix is cleaved enzymatically, which can lead to biofilm dispersion [84].

The extracellular components of *P. aeruginosa* play a significant role in the initial step of biofilm formation. Flagella and type IV pili are essential components of the matrix, have a role in adhesion to the surface, and are linked with initial attachment during biofilm production [86]. Previous studies confirmed that deletion of type IV pilus and flagellum genes resulted in deficient biofilm formation [86]. Therefore, both type IV pili and flagella-dependent motilities seem to have a role in the formation of *P. aeruginosa* biofilm [87,88]. A mutation study determined that the assembly of fimbriae subunits is regulated by a chaperone-usher pathway involved in biofilm formation [89].

Three types of polysaccharides (alginate, Pel, and Psl) are produced by *P. aeruginosa* and contribute to biofilm development by providing structural integrity [90]. The alginate is a linear, unbranched polymer made up of L-guluronic acid and D-mannuronic acid [91]. This provides structural support and protection and is responsible for nutrient and water retention in the biofilm [92]. The Pel polysaccharide is rich in glucose and is involved in pellicle formation [93], while Psl is made up of a pentasaccharide containing D-mannose, L-rhamnose, and D-glucose [94]. Both Pel and Psl provide a structured platform for biofilm production [92]. During the biofilm formation, a subpopulation of cells lyse and release eDNA, an important component of the *P. aeruginosa* biofilm matrix. The eDNA also contributes to cellular alignment, serves as a nutrient source and a cation chelator, and allows the biofilm environment to become acidic, which limits antimicrobial agent penetration [84].

#### 4.1.1. Role of TCSs in Each Stage of Biofilm Formation

Biofilm formation in *P. aeruginosa* is controlled tightly by TCSs. TCSs control the production of key components of the biofilm in response to environmental stimuli and ultimately trigger the bacterium to change from planktonic to sessile life phases, and vice versa. Figure 3 illustrates the critical TCSs and genes responsible for each stage of biofilm formation.

Reversible or initial attachment is an important stage in biofilm formation. Many TCSs are responsible for surface attachment. Important surface components, including flagella, type IV pili, and fimbriae, are responsible for motility and initial attachment. The promoter fusions and microarray studies revealed that *fleSR* transcriptional activation is directly regulated by a master transcriptional regulator FleQ [40]. Furthermore, at least 26 genes in *P. aeruginosa* are regulated directly or indirectly by FleSR [40]. Among these genes, approximately 20 are responsible for flagellar formation, which eventually facilitates reversible attachment during biofilm formation. Consistently, *P. aeruginosa* carrying *fleS* and *fleR* mutations have shown a significant reduction in bacterial adherence to the substrate [95,96]. Interestingly, it has been noted that FleQ regulates the *fleSR* operon [40]. PilSR is another important TCS responsible for regulating the expression of the type IV pilus and facilitating initial attachment through twitching motility [41]. Earlier mutational studies have shown that *pilR* and *fleR* genes are necessary for twitching and swimming motility, respectively [97]. Roc is another TCS in *P. aeruginosa* and is comprised of RocS1 and RocA1, a sensor kinase and RR, respectively. Roc is another TCS in *P. aeruginosa* and comprised of RocS1, RocR and RocA1, which is a sensor HK, an antagonist of RocA1, and RR, respectively. The Roc stimulates *cupB* and *cupC* gene expression, which leads to CupB and CupC fimbriae production, responsible for adhesion. A two-hybrid assay revealed that RocS1 interacts with both RocA1 and RocR, which suggests that RocR modulates RocA1 activities by competing with RocA1 for the interaction with RocS1 [42]. These gene clusters in the PA14 strain are responsible for biofilm maturation [78]. Other TCSs, namely FimS-AlgR (AlgZ-AlgR) and KinB-AlgB, play a role in motility regulation and positively regulate alginate biosynthesis [43,44]. AlgB RR directly binds to the *algD* promoter, which is a key gene for alginate biosynthesis [45], which is crucial for *P. aeruginosa* biofilm development and has a role in adherence [98]. An earlier study showed that AlgR RR positively regulates the transcription of *algD* [99]. A previous study examined the role of BfiSR, another TCS, in biofilm formation and observed that it regulates the irreversible attachment of biofilm through transcriptional activation of *cafA* that encodes RNaseG [47]. Deactivation of the *cafA* gene leads to enhancement of the RsmZ level and inhibits biofilm formation. Rsm (repressor of secondary metabolism) system is a well-characterized small RNA (sRNA)-based regulatory system consisting of Rsm X, Y, and Z. Upregulation of *cafA* restored biofilm formation in the mutant *bfiS* background and decreased the level of *rsmZ* with respect to that of wild-type PA14 [47]. Among many TCSs in the *P. aeruginosa* genome, GacSA is responsible for switching from acute to chronic infection by controlling the expression of small RNAs (sRNAs), *rsmY and rmsZ*, through interaction with RetS and LadS [48,49]. It was suggested that LadS and RetS act agonistically and antagonistically, respectively, by forming a hetero complex with GacS. PA1611, a hybrid HK, also affects the biofilm formation by forming a heterocomplex with RetS [48]. A sensor HK GacS detects the unknown environmental signals which trigger the transfer of phosphate to RR GacA, which controls the production of sRNAs, such as RsmY and RsmZ. These RNAs regulate biofilm formation, motility, and T3SS via direct or indirect mechanisms [51]. RsmY and RsmZ bind to the RsmA regulatory protein and build an RsmY/Z-RsmA complex. This complex enhances the levels of *pel* and *psl* operons that are responsible for formation of microcolonies in the initial and later stages of biofilm formation in *P. aeruginosa* PA01 strain [23,49]. A previous report has suggested that PvrSR and RcsCB are similar to the Roc system and are responsible for *cupD* regulation [52]. Two additional TCS, PvrS and RcsC, are hybrid and unorthodox sensor histidine kinases, respectively. RcsB RR stimulates *cupD* expression, while PvrR RR has antagonistic activity to that of RcsB on *cupD* expression. PvrR is involved in the c-di-GMP degradation pathway through phosphodiesterase activity [52]. Pyruvate fermentation and a response regulator, MifR, support the formation of a microcolony [100]. Another study confirmed that MifSR senses the levels of α-ketoglutarate (α-KG) and regulates its transport and metabolism [53]. The activated MifR, along with sigma factor RpoN (σ^54^), initiates the transcription of the *PA5530* gene, which is an α-KG-specific transporter gene. However, the exact mechanism of action to form the microcolonies is not known [53].

#### 4.1.2. TCS Involvement in Controlling Biofilm Formation

Mutations in *bfiS*, *bfmR*, and *mifR* genes prevent biofilm formation, indicating involvement of these genes in various stages of biofilm formation. Interruptions in sequencing and assembly of these genes disturb the overall development and maintenance of the biofilm [101]. BfmR activates the *phdA* gene and has a role in the release of eDNA, which provides integrity in biofilm maturation [54]. PprAB is an another important TCS that plays a role in stimulating *cupE* gene cluster expression and producing CupE fimbriae that is responsible for cell-to-cell connections during microcolony formation, and thus is involved in colony formation in the early stages of biofilm formation and, also in 3D mushroom-like structure shaping during the biofilm maturation [55]. The production of Flp pilin, a major subunit of type IVb pili responsible for adhesion, can be observed in the late stationary phase. PprB is a RR that binds to three intergenic regions upstream of the *flp–rcp*, *tadF–fppA*, and *pprB* genes [102]. Post-maturation and biofilm dispersion are necessary stages for biofilm persistence. The detached cells migrate away, which accounts for reversible growth, and ultimately help bacteria to maintain the biofilm [84]. A mutation study on *bqsS* has demonstrated that BqsSR plays a critical role in biofilm degeneration by modulating the synthesis of rhamnolipids and signaling molecules such as 4-hydroxy-2-heptylquinoline (C4HSL) and pseudomonas quinolone signal (PQS) [56]. Production of rhamnolipids is controlled by the *rhlAB* operon.

### 4.2. Motility System

The motility system is an important virulence factor in many pathogenic bacteria because it is necessary for proliferation, colonization, and infection. Motility allows bacteria to adjust to diverse environmental conditions [103]. The three distinct types of motility in *P. aeruginosa* are swimming, swarming, and twitching [104].

#### 4.2.1. Role of TCSs in Swimming and Swarming Motilities 

*P. aeruginosa* possesses a single polar unsheathed flagellum that is crucial for both swimming and swarming motility [105,106]. Flagellar proteins are also responsible for adhesion, invasion, and biofilm formation [107]. Flagellin, a flagellar protein, facilitates the inflammatory response via the innate immune system and interacts specifically with a number of pattern recognition receptors (PRRs) of the host.

**Figure 4:**
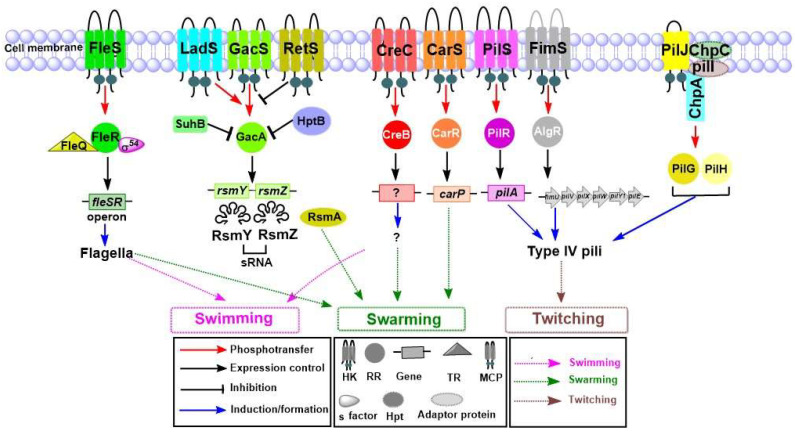
Functional characterization of TCSs in *P. aeruginosa* and their involvement in motility-related virulence. The FleS sensor HK phosphorylates its cognate RR FleR and facilitates transcription of flagellar genes. The interconnected transcriptional regulatory circuits consisting of FleR, FleQ TR, and σ^54^ factors have a major role in flagellar biogenesis and are responsible for swimming and swarming motility. GacSA works along with hybrid sensor HKs LadS and RetS through a parallel and antagonistic mechanism. The free RsmA regulatory protein is involved in swarming motility. The SuhB regulator indirectly regulates motility through the GacSA, HptB also works along with GacSA and indirectly controls free RsmA. CreCB plays a major role in swarming motility by an unknown mechanism. CarSR is involved in swarming motility through its target *carP*. PilSR has a major role in controlling type IV pili and twitching motility. FimS-AlgR also is important for twitching motility. The Chp chemosensory system impacts type IV pili. The MCP receptor PilJ senses the environmental signal and phosphorylates ChpA using two adaptor proteins, ChpC and PilI. Phosphorylated PilG and PilH play a role in the extension and retraction of type IV pili.

Several TCSs are engaged in the synthesis, assembly, and regulation of flagellar proteins, as shown in Figure 4. In *P. aeruginosa*, FleSR is responsible for the expression of many genes involved in flagellar biosynthesis [40]. It is well established that transcriptional and post-transcriptional events are controlled by the interlinked transcriptional regulatory circuits that consist of FleR RR, FleQ (TR), and sigma factor RpoN (σ^54^) [40]. Nonmotile mutants have been found in a large population of *P. aeruginosa*, isolated from CF patients [108]. Previous reports have suggested that restricted motility causes aggregated growth of bacteria, which strikingly increases the resistance of bacteria to macrophage ingestion. Furthermore, it was also found that the non-motile *P. aeruginosa* with aggregated growth has higher antibiotic tolerance as compared to the motile strains [108,109]. The σ factor AlgT has a role in *P. aeruginosa* motility, and its suppression decreases the flagellum expression by preventing the expression of flagellar regulator FleQ [110]. The flagella-impaired strains that have been isolated from CF patients are more pathogenic toward the host immune system than are other motile strains [110]. FleQ regulates the transcription of *fleS-fleR*, as well as a number of additional flagellar, adhesion, and biofilm-associated genes. In addition, several flagellar genes controlled by FleQ are antagonized by the cyclic diguanylate (c-di-GMP)-related pathway [57,58]. GacSA, which works in parallel to and antagonistic with LadS and RetS, also has an important role in motility. Swarming motility, a physiological phenomenon defined as multicellular, flagella-mediated migration of bacteria on a surface. Swarming is regulated by the GacSA signaling system. The sensor proteins and LadS activate the GacSA signaling system, while RetS represses its activation in response to external stimuli. Upon activation, GacSA stimulates the generation of two sRNAs, RsmY and RsmZ, which sequester the free RsmA by forming a RsmA-RsmY-RsmZ complex. Since RsmA has a role in repressing genes involved in chronic infection, such as *pel* and *psl* (biofilm formation), *phz* (pyocyanin), and genes in T6SS; as well as in activating genes associated with acute infection, such as gene sin swarming motility, *lipAH* (lipase), *rhlAB* (rhamnolipids), and genes in T3SS, the GacSA signal triggers the chronic infection via the production of RsmY and RsmZ [59]. Additionally, HptB, a histidine phosphotransfer protein, indirectly controls the expression of RsmA through a poorly-defined mechanism [111]. HptB also has a role in swarming since mutations in *hptB* genes lead to swarming deficiencies [112]. A prior study found that SuhB, a ribosome-associated protein, regulates multiple virulence factors that are responsible for swimming motility, biofilm production, type III secretion, and type VI secretion [113]. SuhB regulates the motile-sessile transition by inversely controlling the swimming motility and biofilm formation through the GacA-RsmY/Z-RsmA system [113]. The same study has revealed that the motility loss in *suhB* mutant strains can be recovered by mutations in *gacA or rsmY*/*Z.* Furthermore, yet another finding in this study was that the excessive production of RsmA protein in *P. aeruginosa* can also rescue the motility defect caused by a mutation in *suhB*. The mutational analysis has revealed that *gacA* or sRNAs *rsmY/rsmZ* or RsmA overproduction are rescued the motility defects in *suhB* mutant [113]. CreCB is an important conserved signaling system in many bacteria including *P. aeruginosa* and plays an important role in swarming and antibiotic resistance [60]. One study demonstrated that CarS HK is responsible for sensing external Ca^2+^ concentration and modulating Ca^2+^ homeostasis via CarR RR. CarSR also regulates swarming motility through a target gene *carP* [61]. PilSR, which plays a role in pilus-dependent twitching motility, is also associated with flagellum-dependent swimming motility [41].

#### 4.2.2. Role of TCSs in Twitching Motility 

*P. aeruginosa* uses hair-like appendages, known as type IV pili, which are an essential virulence factor [114]. Pili are surface organelles important for biofilm initiation, colonization, bacterial aggregation, twitching, and cellular invasion. Most type IV pili use cytoplasmic ATPase to elongate and retract, which are important features of motility [114]. Various TCSs are responsible for production, function, and control of type IV pili in *P. aeruginosa*. Previous research has shown the diversity of a type IV pilin allele based on *P. aeruginosa* strains collected from CF patients and the environment [115]. One report showed that more than 40 *P. aeruginosa* genes contribute to the function of type IV pili [66]. These genes encode major structural protein PilA and other minor proteins such as PilE, PilV, PilX, PilW, PilY1, PilY2, and FimT, which are responsible for formation of the tip and base of the pili [62]. Additionally, other proteins regulate the production of pili and are responsible for twitching motility in response to environmental signals, as shown in Figure 4. PilSR is the most important TCS responsible for expression of the gene *pilA* [63]. The role of FimS-AlgR in the regulation of twitching motility has been explained in earlier studies [65,66]. FimS-AlgR controls the twitching motility by positively regulating the expression of the genes involved in the assembly of minor pilins FimU-PilVWXE and the putative adhesin PilY1 prime pilin, which are known to mediate the twitching motility in *P. aeruginosa* [64]. The twitching motility of *P. aeruginosa* is controlled by a chemosensory system, Chp system [67]. The putative HK ChpA is coupled with a methyl-accepting chemotaxis protein (MCP) receptor, PilJ, which is controlled by two CheW-like adaptor proteins, ChpC and PilI. This complex senses currently unknown environmental signals, and facilitates the conformational change of PilJ, which causes ChpA autophosphorylation [67]. Moreover, it was proposed that PilJ also recognizes the major pilin subunit PilA as a sensor of mechanically induced conformational changes in the stretched type IV pili [116]. The phosphorylated RR PilG interacts with the motor complex of PilZ, ATPase PilB, and diguanylate cyclase FimX to facilitate pilus extension, while another RR PilH plays a role in retraction of type IV pili through interaction with ATPases PilT and PilU [68].

### 4.3. Pyocyanin 

Pyocyanin is a blue-green pigment with a strong antibiotic effect against other bacterial species [117]. Several infections associated with pyocyanin cytotoxic effects have been reported, and they involve pro-inflammatory and free radical production resulting in cellular damage and necrosis [118,119,120]. Pyocyanin is produced in both the planktonic and biofilm states. However, since it plays a role in biofilm formation in *P. aeruginosa* [121], pyocyanin detection can be employed as a rapid approach for detecting *P. aeruginosa* infections in patients [122].

#### TCSs Responsible for Pyocyanin Production

**Figure 5:**
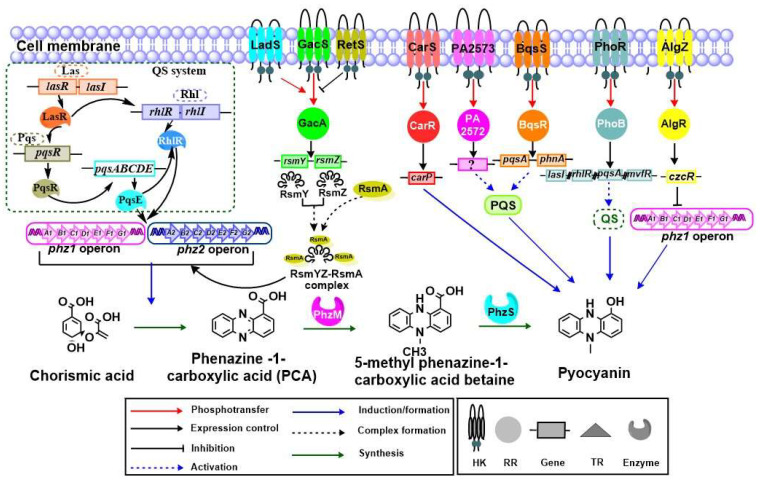
Biosynthesis and regulation of pyocyanin pigment by TCSs. The QS system (las, rhl, and pqs), the autoinducer synthase (PqsE), and RhlR TR are highlighted in a green dotted rectangle and play an important role in regulation of *phz* genes. Both *phz1* and *phz2* operons are responsible for biosynthesis of pyocyanin. They convert chorismic acid into phenazine-1-carboxylic acid (PCA), which is converted into 5-methyl phenazine-1-carboxylic acid betaine and finally pyocyanin pigment via phenazine enzymes (PhzM and PhzS). GacSA along with LadS and RetS hybrid HKs function in a parallel and antagonistic approach. The RsmY/Z-RsmA complex enhances the regulation of *phz* genes. CarSR is responsible for pyocyanin production by controlling the *carP.* PA2572-PA2573 and BqsSR also are responsible for pyocyanin production through PQS. PhoRB is another important TCS that is responsible for pyocyanin production through the QS network. AlgZR plays an antagonistic role in the production of pyocyanin.

Like many other virulence factors, pyocyanin is controlled by a complex TCS network, as shown in Figure 5. Pyocyanin allows the bacterial population to coordinate a response to an environmental change [123]. Pyocyanin production is regulated by three interlinked QS systems *las*, *rhl*, and *pqs* [124,125,126]. Moreover, thioesterase (PqsE), 2-heptyl-4-hydroxyquinoline (HHQ), and 2-heptyl-3-hydroxy-4-quinolone (PQS) play essential roles in pyocyanin production [127,128]. In *P. aeruginosa*, the PQS QS system is regulated positively and negatively by Las and Rhl, respectively, and is responsible for pyocyanin synthesis [129,130]. A previous report has suggested that *phz1* and *phz2* operons play a central role in the biosynthesis of pyocyanin. Both *phz1* and *phz2* operons consist of functionally-associated genes, *phzA1B1C1D1E1F1G1* and *phzA2B2C2D2E2F2G2*, respectively, and each operon encodes a set of enzymes responsible for the synthesis of phenazine-1-carboxylic acid from its precursor chorismic acid. *P. aeruginosa* produces pyocyanin through multiple steps that begin with chorismic acid production via a complex phenazine biosynthetic route [131,132]. PCA is transferred to pyocyanin with the help of an adenosylmethionine-dependent methyltransferase (PhzM) and flavin-comprising hydroxylase (PhzS) [133,134].

LasR positively regulates the expression of *rhlR* and *pqsR* while RhlR inhibits the expression of *pqsABCDE*. PqsE activates the Rhl system by an unknown mechanism and this system directly controls the production of pyocyanin [135,136]. Pseudomonas quinolone system (PQS) system consists of five genes. *pqsABCDE* in the chromosome of *P. aeruginosa*, PqsA, an anthranilate-coenzyme A ligase, plays a role in the first step of PQS biosynthesis by triggering the synthesis of anthranilate-coenzyme A. PqsD controls the production of 2-aminobenzoylacetate (2-ABA) from anthraniloyl-coenzyme A and malonyl-coenzyme A. PqsB and PqsC form a heterodimer that catalyzes a reaction to produce 2-heptyl-4-quinolone (HHQ) through the condensation of octanoyl-coenzyme A and 2-ABA. Finally, PqsE thioesterase in alkylquinolone biosynthesis hydrolyzes the biosynthetic intermediate to generate 2-ABA [124].

GacSA in *P. aeruginosa* also works with RetS hybrid sensor HKs that are associated with the production of phenazine metabolites [137]. Previous reports suggested that CarSR is responsible for sensing the external Ca^2+^ concentration to modulate Ca^2+^ homeostasis [61]. Furthermore, the same studies also suggested that CarSR regulates the transcription of *carP*, which modulates the pyocyanin production and swarming motility in the high Ca^2+^ condition. Additionally, an orphan chemotaxis sensor, PA2573, regulates the production of pyocyanin in *P. aeruginosa*. Consistently, it was observed that the mutation in the PA2573 gene significantly reduces the production of pyocyanin [69]. Another study reported that pyocyanin production also is controlled by BqsSR that is positively regulated by the PQS system [56]. A gene expression study explained the role of BqsSR in modulating the transcriptional expression of *pqsA* and *phnA*, which are responsible for PQS biosynthesis [56]. Accordingly, it was reported that mutations in *bqsS* and *bqsR* significantly decrease pyocyanin production [56]. One study observed that the production of pyocyanin was affected by a RR and PhoB under phosphate-limited conditions [70]. PhoB is responsible for activation of genes involved in QS, such as *lasI*, *rhlR*, *pqsA*, and *mvfR* [70]. AlgZR is a key component that regulates pyocyanin production through CzcR, a repressor of pyocyanin. CzcR directly binds to the *phz1* operon to repress the synthesis of pyocyanin [71]. 

### 4.4. Secretory System and Secreted Virulence Factors

The secretory system and secreted virulence factors of *P. aeruginosa* are considered to be essential. The different types of secretion systems (Types I, II, III, V, and VI) identified in *P. aeruginosa* participate in pathogenicity by secreting a variety of toxins and hydrolytic enzymes [138].

#### 4.4.1. Secretory System 

Of these secretion systems, type II and III (T2SS, and T3SS) are important. The T2SS of *P. aeruginosa* is responsible for secreting various secretory proteins such as lipase, phospholipase, exotoxin A, proteases, and alkaline phosphatase. It is well known that T2SS secretes proteins into the extracellular environment using a pilus-like structure [107]. Another type of T2SS, which is denoted as the third Xcp homolog (Txc), was discovered in *P. aeruginosa* strain PA7 [72]. A protein secreted through the Txc secretion system binds to chitin and thus it is known as chitin-binding protein E (CbpE). Apart from CbpE, other chitin-binding proteins such as CbpD (chitin-binding protein D) and elastase are also secreted through the T2SS system in *P. aeruginosa* [139]. Most of these secretory proteins promote the virulence of *P. aeruginosa* by damaging host cells and tissues. Among the proteins secreted by the T2SS, exotoxin A (also known as ToxA), is an important secretory protein. ToxA possesses ADP-ribosylation activity that helps bacteria to alter the protein synthesis of host cells [140]. Elastase is another virulence factor that belongs to the protease family. A previous review has extensively discussed the role of elastase in the modulation of initial defense mechanisms which eventually led to damaging host tissues [141]. Earlier research demonstrated that the LasA protease and LasB elastase play important a role in the pathogenesis of *P. aeruginosa* [142].

The T3SS is another important secretion system associated with higher mortality and cytotoxin delivery to the host cell [143]. Early research demonstrated that *exoS*, *exoT*, *exoU*, and *exoY* genes can be used as markers for chronic *P. aeruginosa* infection in hospitals [132]. T3SS plays a critical role in toxin secretion, host tissue destruction, and host immune response disruption. There are four major toxins or effector proteins controlled by the T3SS: ExoS, ExoT, ExoU, and ExoY [36]. ExoS exhibits GTPase-activating protein activity (GAP) and ADP ribosyl transferase activity (ADPRT), while ExoT exhibits N-terminal GAP activity and carboxy-terminal ADPRT activity [144]. ExoS and ExoT are considered bifunctional cytotoxins. ExoU has a role in acute infection and is associated with oxidative imbalance by acting as an important phospholipase [145]. ExoY is an adenylate cyclase that has a role in breaking down host microtubules and in disrupting the cell to cell junction in endothelial cells, which eventually leads to tissue edema [146].

#### 4.4.2. TCSs That Influence the Secretion Systems and Their Substrates

**Figure 6:**
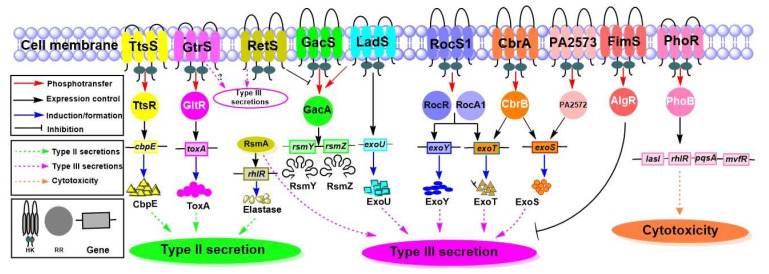
TCS-dependent regulation of secreted proteins. This figure summarizes important sensor HKs and their cognate RRs that are responsible for proteins secreted by type II, type III, and Txc secretion systems. The TtsSR is responsible for the Txc secretion and is involved in the production of CbpE. GtrS-GltR is responsible for the production of ToxA, which is an important toxin secreted by the type II secretion system. GacSA interacts with LadS and RetS in agonistic and antagonistic manners, respectively. GacSA controls both elastase and type III secretion systems by producing RsmY and RsmZ, which prevent the activities of RsmA by forming a RsmA-RsmY-RsmZ complex. LadS, specifically, is involved in regulation of ExoU. The RocS1-RocR-RocA1 is involved in the production of ExoY and ExoT toxins secreted by the type III secretion system. CbrAB has a role in the synthesis of ExoS and ExoT toxins. The PA2573-PA2572 is involved in the synthesis of the ExoS toxin, and the FimS-AlgR is responsible for suppressing type III secretion. The PhoRB is required for cytotoxicity under a phosphate-limited condition.

Many TCSs are involved in the regulation of the secreted proteins in T2SS and T3SS (Figure 6) [72]. An earlier study revealed that GltR regulates the expression of genes for glucose metabolism and transport [73], while sensor kinase GtrS facilitates bacterial host interaction and dissemination [147]. However, it was revealed that GtrS and GltR forms a TCS that is responsible for regulating *toxA* gene expression [73]. The role of GtrS HK in type III secretion has been elucidated in response to host cells. In the pneumonia infection model, GtrS was found critical for the host cell response during colonization and dissemination. It was also revealed that GtrS induces T3SS under microaerobic conditions [148].

GacSA regulates type III secretion through RsmA activation via an unknown environmental stimulus [74]. The hybrid sensor kinase PA1611 interacts with RetS and facilitates type III secretion and biofilm formation [51,97]. A previous study showed that marine strain *P. aeruginosa* ID4365 modulates the production of elastase, rhamnolipids, and pyocyanin via RhlR and RsmA [76]. This report also suggested that inactivation of *rsmA* causes a surge in pyocyanin production but decreases elastase and rhamnolipid production via reduction of RhlR. Based on the mutational study of LadS, it was proposed that ExoU is regulated by LadS in *P. aeruginosa* at the transcriptional level, as well as the protein or phenotypic level via an unknown mechanism [77]. Another study explained that the SadARS signaling system, also called the RocS1−RocR−RocA1 system, contributes to production of secreted proteins through the type III secretion system [19,78]. A microarray study revealed that SadARS has a role in the synthesis of ExoY and ExoT [78]. CbrAB plays a role in the regulation of type III secretion and its effector exoenzymes ExoS and ExoT [79]. Another study explained the role of PA2573-PA2572 in the production of the ExoS toxin [69]. PhoRB is responsible for the expression of various virulence genes involved in cytotoxicity through QS system [70,148]. Among the key virulence factors regulated by QS, *lasI*, *pqsA*, *mvfR*, and *rhlR* are activated by PhoB in a phosphate-limiting condition [70]. A previous study indicated that FimS-AlgR plays an important role in the type III secretion since AlgR RR suppresses the type III secretion system in a mucoid background [149]. During the chronic *P. aeruginosa* CF infection, the strains switched into an alginate-overproducing mucoid phenotype, which protects them from host immune responses and oxidative burst [150,151]. TtsSR positively regulates the expression of *txc* gene clusters and the *cbpE* gene [72].

## 5. TCSs as Targets for Drug Development

Rapid emergence of antimicrobial resistance has become a major health threat worldwide. Therefore, developing new antibiotics and other treatment avenues are critical. However, there are limited numbers of targets for drug development. In this aspect, it is important to understand the structures and functions of TCSs to use them as new drug targets based on their role in production of virulence factors and antibiotic resistance [19]. Numerous studies have shown that TCSs are necessary for the coordinated expression of virulence determinants. They are important for the bacterial growth and survival. TCSs of *P. aeruginosa* have a complex signaling mechanism that is involved in host pathogenesis. The regulatory behavior of TCSs makes them excellent targets for antimicrobial therapy to overcome infection caused by drug-resistant bacteria [152,153]. Many studies have described that both antibiotics and anti-virulence drugs can be designed by targeting TCSs [154]. The wide dominance and functional variety of TCSs also favor the possibilities of screening novel small-molecule inhibitors against at least one of these TCSs [152].

TCSs play a central role not only in the coordination of numerous virulence factors, but also in bacterial growth and viability. Moreover, histidine phosphorylation acts as a key signaling mechanism in the bacterial TCSs while Ser/Thr/Tyr phosphorylation occurs during the cellular signal transduction in eukaryotic cells. Therefore, bacterial TCSs are considered to be promising targets for the development of novel antibiotics or antivirulence agents [152]. There have been reports on the development of antibiotics against *P. aeruginosa*, through the targeting of TCSs. For example, halogenated phenyl-thiazoles were developed to be used as small molecule inhibitors of AlgR2 TCS. However, these compounds also showed inhibitory activity on KinA, CheA, and NRII [155]. In another report, it was proposed that mucin glycans can be used for controlling the *P. aeruginosa* infection by inhibiting GacS-GacA via RetS-dependent signaling [156]. These promising outcomes signify the role of TCSs in developing anti-virulent or antimicrobial drugs. Despite such a significant role, there are several issues to be considered for the development of TCS-targeting inhibitors. For one, the development of a drug that is highly selective to TCSs may be difficult due to the high level of structural similarities among bacterial HKs and RRs [157,158,159]. Furthermore, the toxicity issue poses an additional barrier to the development of TCS-targeting drugs. Since the ATP-binding pocket in bacterial TCSs is sequentially and structurally similar to those in some human proteins, drugs targeting bacterial TCSs may show inhibitory activities on human proteins [160].

## 6. Conclusions

*P. aeruginosa* is a Gram-negative bacterium, and its virulence is associated with TCSs. Many TCSs directly regulate *P. aeruginosa* virulence, including biofilm formation, motility, toxin secretion, and pigment production. Control of important bacterial TCSs offers a new opportunity for treatment of bacterial infections. Many TCSs are responsible for regulation of biofilm formation in a stage-specific manner, leading to chronic infection. Moreover, a number of TCSs is involved in motility-related phenotypes, pyocyanin biosynthesis, and cytotoxin secretion. These complex TCSs allow bacteria to react suitably to diverse environmental stimuli. Additionally, TCSs undergo substantial selective pressure within hosts, mainly during acute and chronic infection. Therefore, it is essential to understand how TCSs detect environmental signals, transduce signals, and regulate gene expression during the infectious process to increase our knowledge on bacterial pathogenicity controlled by TCSs. Further studies on virulence factors and their cognate TCSs will contribute to developing innovative antibacterial and anti-virulence strategies to overcome AMR.

## Figures and Tables

**Table 1:** Summary of genome size and TCSs of *P. aeruginosa* with other Gram-negative pathogens.

Bacterial Strain	Genome Size (Mbp)	Number of Genes (NCBI Ref Seq No.)	Sensor Kinase	Response Regulator	Reference
*Pseudomonas aeruginosa* PA01	6.26	5697 (NC_002516.2)	63	64	[18,24]
*Escherichia coli* K12	4.64	4609 (NC_000913.3)	28	32	[25,28]
*Klebsiella pneumoniae* HS11286	5.33	5404 (NC_016845.1)	32	32	[23,26]
*Acinetobacter baumanni* XH386	4.09	4062 (CP010779.1)	14	15	[23,27]

**Table 2:** List of TCSs that contribute to virulence in *P. aeruginosa*.

Virulence	Description	Gene	Virulence Factor	TCS	Reference
Biofilm	Reversible attachment/adherence	*fleSR* operon	Flagella	FleSR	[40]
		*pilA*	Type IV pili	PilSR	[41].
		*cupB*, *cupC*	CupC fimbriae	RocS1-RocR-RocA1	[42]
	Irreversible attachment	*algD*	Alginate	FimS-AlgR, KinB-AlgB	[43,44,45,46]
		*cafA*	Rnase G	BfiSR	[47]
	Microcolony	*psl*, *pel*	Exopolysaccharides (Pel, Psl)	GacSA, RetS	[23,48,49,50,51]
		*cupD*	CupD fimbriae	RcsCB, PvrSR	[52]
		*PA5330*		MifSR	[53]
	Maturation	*phdA*	eDNA	BfmSR	[54]
		*cupE*	Cup E fimbriae	PprAB	[55]
	Dispersion	*rhlAB* operon	Rhamnolipid	BqsSR	[56]
Motility	Swimming/ Swarming	*fleSR* operon	Flagella	FleSR	[40,57,58]
				GacSA	[59]
				CreCB	[60]
		*carP*		CarSR	[19,61]
	Twitching	*pilA*	Type IV pili	PilSR	[41,62,63]
		*fimU*, *pilV*, *pilW, pilX, pilE*, *pilY1*		FimS-AlgR	[64,65,66]
		*PilT*, *pilU*, *fimX, pilB*, *pilZ*		ChpA-PilG	[67,68]
Pigment	Pyocyanin	*phz*	Pyocyanin	GacSA-LadS-RetS	[59]
		*carP*		CarSR	[61]
		*pqsA*, *phnA*		BqsSR	[56]
				PA2573-PA2572	[69]
		*lasI*, *rhlR*, *pqsA*, *mvfR*		PhoRB	[70]
		*czcR*		AlgZR	[71]
Toxin	Secreted by Type II, Type III secretion systems	*cbpE*	CbpE	TtsSR	[72]
		*toxA*	ToxA	GtrS-GltR	[73]
			Type III secretion	GacS-LadS-RetS, CsrA/RsmA	[74,75]
		*rhlR*	Elastase	RsmA	[76]
		*exoU*	ExoU	LadS	[77]
		*exoY*, *exoT,*	ExoY, ExoT	RocS1-RocR-RocA1	[78]
		*exoT*, *exoS*	ExoT, ExoS	CbrAB	[79]
		*exoS*	ExoS	PA2573-PA2572	[69]
		*lasI*, *rhlR*, *pqsA*, *mvfR*	Cytotoxicity	PhoRB	[70]
		*algD*		FimS-AlgR	[19,46]

## Data Availability

Not applicable.
